# Linear Integration of Tactile and Non-tactile Inputs Mediates Estimation of Fingertip Relative Position

**DOI:** 10.3389/fnins.2019.00068

**Published:** 2019-02-11

**Authors:** Simone Toma, Daisuke Shibata, Francesco Chinello, Domenico Prattichizzo, Marco Santello

**Affiliations:** ^1^School of Biological and Health Systems Engineering, Arizona State University, Tempe, AZ, United States; ^2^Athletic Training Education Program, Department of Health Exercise and Sports Sciences, University of New Mexico, Albuquerque, NM, United States; ^3^Department of Information Engineering, University of Siena, Siena, Italy; ^4^Department of Business Development and Technology, Aarhus University, Aarhus, Denmark

**Keywords:** perception, haptic bias, digit forces, tactile inputs, digit position sense

## Abstract

While skin, joints and muscles receptors alone provide lower level information about individual variables (e.g., exerted limb force and limb displacement), the distance between limb endpoints (i.e., relative position) has to be extracted from high level integration of somatosensory and motor signals. In particular, estimation of fingertip relative position likely involves more complex sensorimotor transformations than those underlying hand or arm position sense: the brain has to estimate where each fingertip is relative to the hand and where fingertips are relative to each other. It has been demonstrated that during grasping, feedback of digit position drives rapid adjustments of fingers force control. However, it has been shown that estimation of fingertips' relative position can be biased by digit forces. These findings raise the question of how the brain combines concurrent tactile (i.e., cutaneous mechanoreceptors afferents induced by skin pressure and stretch) and non-tactile (i.e., both descending motor command and joint/muscle receptors signals associated to muscle contraction) digit force-related inputs for fingertip distance estimation. Here we addressed this question by quantifying the contribution of tactile and non-tactile force-related inputs for the estimation of fingertip relative position. We asked subjects to match fingertip vertical distance relying only on either tactile or non-tactile inputs from the thumb and index fingertip, and compared their performance with the condition where both types of inputs were combined. We found that (a) the bias in the estimation of fingertip distance persisted when tactile inputs and non-tactile force-related signals were presented in isolation; (b) tactile signals contributed the most to the estimation of fingertip distance; (c) linear summation of the matching errors relying only on either tactile or non-tactile inputs was comparable to the matching error when both inputs were simultaneously available. These findings reveal a greater role of tactile signals for sensing fingertip distance and suggest a linear integration mechanism with non-tactile inputs for the estimation of fingertip relative position.

## Introduction

The central nervous system (CNS) has a remarkable ability to rapidly integrate sensory feedback of digit position with motor commands for force control (Fu et al., 2010, 2011). Furthermore, it has been shown that successful manipulation can be attained also in the absence of feedback of digit position associated to motor commands responsible for digit placement, or vision of the fingertips (Jenmalm and Johansson, 1997; Voudouris et al., 2012; Fu and Santello, 2014). This evidence supports the hypothesis that, during hand-object interactions, the CNS integrates available sensorimotor inputs into an accurate representation of the hand and fingers in space (Longo et al., 2010; Butler et al., 2015). While the role of afferent signals in manipulation has been extensively investigated (see Johansson and Flanagan, 2009 for a review), little is known about how these signals are combined with motor commands to estimate the fingertip position relative to each other (e.g., the thumb relative to the index finger).

Due to the lack of dedicated sensory receptors encoding distance between limb endpoints, in absence of vision, estimation of fingertip position requires processing of information conveyed by at least three different sensory or sensorimotor inputs: (a) tactile cues arising from mechanical deformation of the finger pads; (b) proprioceptive inputs triggered by changes in length and tension of the muscle-tendon complex, and joint angle; (c) and a copy of the motor command responsible for fingers placements and contact forces, i.e., efference copy. Cutaneous receptors can provide information about the magnitude and direction of force acting on the finger pad (Birznieks et al., 2001; Jenmalm et al., 2003; Panarese and Edin, 2011; see Johansson and Flanagan, 2009 for a review). This ability to encode force-related inputs allows the CNS to gather exteroceptive cues necessary to estimate object features, e.g., object compliance, size, and hand configuration (Longo et al., 2010). Similarly, proprioceptive inputs are known to influence arm and hand position sense when tactile inputs are eliminated or reduced (Ribot-Ciscar et al., 2003; Proske and Gandevia, 2012). Finally, the role of motor commands for estimation of relative finger position has been explored in our previous work by asking subjects to perform a digit position matching task after exerting large normal and tangential forces against a contact surface, thus involving tactile input, proprioceptive input, and efference copy (Shibata et al., 2014). We found that subjects consistently overestimated fingertip vertical distance when digit forces of the thumb and index fingertip were exerted in opposite directions. Specifically, the thumb was placed higher than the index fingertip when thumb and index finger tangential forces were directed upward and downward, respectively, and vice versa. This suggests that the efference copy overrode the information provided by afferents signals about fingertips' relative position. This result also raised important questions regarding the CNS' limited ability to integrate feedback from tactile and proprioceptive inputs with efference copy, and their relative contribution to the estimation of fingertip distance.

In the present study, we address these questions by investigating the relative role of tactile and non-tactile inputs on force-related bias of fingertip distance estimation. As done by other studies (Robles-De-La-Torre and Hayward, 2001; Moscatelli et al., 2016), we leveraged a perceptual illusion as a window into how tactile and non-tactile signals are combined for the perception of digit relative position.

**Figure 1:**
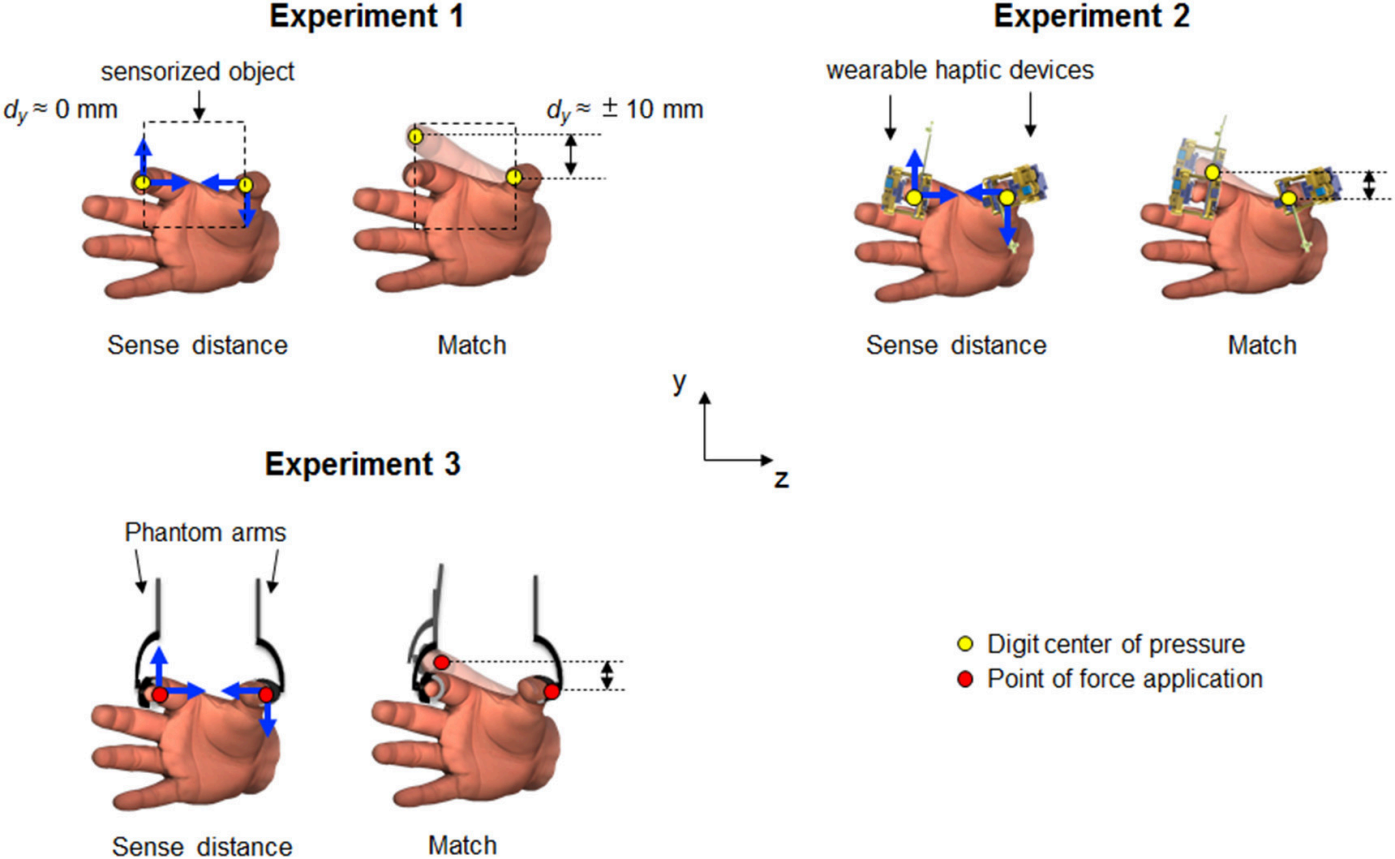
Experimental set-ups. In each experiment, subjects were required to sense and memorize fingertip vertical distance (*d*_*y*_, vertical black double arrows) while either exerting (Experiment 1 and 3) or experiencing (Experiment 2) digit forces (“Sense distance”). Subjects was then asked to reproduce the memorized digit position (“Match”). Direction of force related input associated to voluntary digit forces or passive fingertip stimulation are represented by blue arrows. Opaque fingers depict the hypothetical bias exhibited during reproduction. Yellow and red dots represent digit center of pressure (CoP) for Experiment 1 and 2, and point of force application (PFA) for Experiment 3, respectively. Dashed lines in Experiment 1 depict the static sensorized object. Subjects in Experiment 1 were provided by both tactile and non-tactile inputs during sense distance. In Experiment 2, only tactile signals were available to sense and memorize digit distance. Since the finger-pad was not involved in the task, during Experiment 3 subjects relied only on non-tactile signals to estimate digit distance.

In two experiments, we asked subjects to match fingertip vertical distance by relying only on either tactile (Experiment 2; Figure 1) or non-tactile (Experiment 3; Figure 1) inputs associated with forces from the thumb and index finger. We then compared their matching performance with the scenario where both types of sensory information were combined (Experiment 1; Figure 1). Based on the overestimated fingertip vertical distance found in our previous work (Shibata et al., 2014), we expected fingertip distance estimation bias to occur only in the “opposite forces” condition, with the magnitude of matching errors in each experiment denoting the relative contribution of tactile and non-tactile inputs. Based on the known force-direction encoding properties of tactile afferents in the finger (Birznieks et al., 2001; Johansson and Flanagan, 2009), we expected tactile inputs arising from mechanical deformation of the finger pad to contribute to the misperception of fingertip distance. Similarly, non-tactile inputs are known to play a significant role in the perception of absolute limb position (Proske and Gandevia, 2012) and body representation (Longo et al., 2010). Therefore, we hypothesized that tactile and non-tactile inputs each contribute to fingertip distance estimation. An additional question of our study was to determine the relative contribution of tactile and non-tactile input on fingertip distance estimation and bias.

Our results support our hypothesis while also revealing that tactile inputs contribute the most to the estimation bias. Moreover, the similarity between the bias exhibited in Experiment 1 and the sum of biases observed in Experiment 2 and 3 suggests that the brain linearly combines tactile and non-tactile signals for the estimation of fingertip distance.

## Materials and Methods

### Subjects

Three groups of 10 different subjects participated in three experiments: Experiment 1 (6 females, mean age ± SD: 23.3 ± 6 years.), Experiment 2 (4 females, 27.1 ± 3.8 years.), and Experiment 3 (5 females, 26.3 ± 4.0 years.). Non parametric Kruskal-Wallis ANOVA did not show any statistical differences among subjects' group age, i.e., $\chi_{29}^{2}$ = 4.09, *p* = 0.13. Data from the subjects participating in Experiment 1 were collected and partially presented in a previous report (Shibata et al., 2014). All subjects were right-handed (self-reported) and naïve to the purpose of the study. Subjects gave written informed consent to the procedures approved by Office of Research Integrity and Assurance at Arizona State University (protocol ID: 13020088), in conformity with the Declaration of Helsinki on the use of human subjects in research.

### Apparatus and Procedures

**Figure 2:**
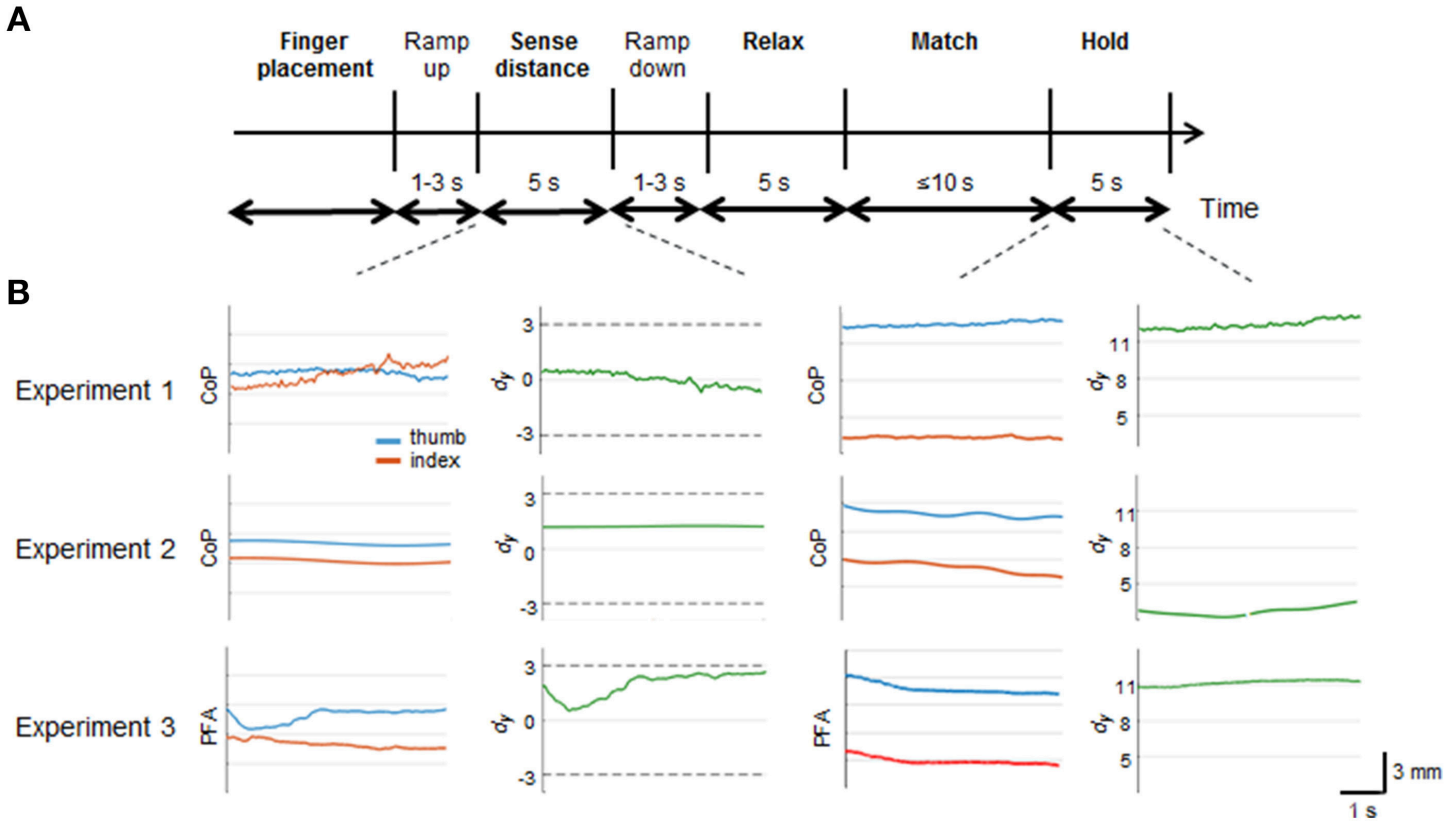
Experimental protocols and trial epochs used in each experiment. **(A)** temporal sequence of the phases characterizing each trial in all experiments. In bold are the main phases. *Ramp up* and *ramp down* represent the time spent by the haptic devices in Experiment 2 and 3 to reach the force threshold and to come back to zero force value, respectively. **(B)** profiles of relative (*d*_*y*_; green traces) and absolute (CoP or PFA) digit finger positions (red and blue traces) from a representative subject. Data are from “*Sense distance*” and “*Hold*” phases of *T*_*UP*_*-I*_*DN*_force combination presented in each experiment. Horizontal dash lines denote the range of *d*_*y*_ subjects experienced during the sense phase. Note the different scale of the vertical axes for *d*_*y*_ during the “Sense distance” and Hold” phases.

All three experiments were composed of 5 main phases (bold in Figure 2A): digit placement in collinear position (“*Finger placement*”); perception and memorization of digit position (“*Sense distance*”); fingers at rest (“*Relax*”); reproduction of memorized digit position (“*Match”*); and maintain digit recalled position (“*Hold*”). Importantly, during the sense distance phase subjects could be provided with force-related tactile and non-tactile inputs associated to either voluntary digit forces (Experiment 3) or external stimulations of the finger pad (Experiment 2), respectively. Such an independent manipulation of the sensorimotor signals available in the sense distance phase aimed to quantify the individual contribution of tactile and non-tactile inputs for finger distance estimation.

#### Experiment 1

The goal of this experiment was to quantify subject's ability to estimate the relative digit position of the thumb and index finger when both tactile and non-tactile inputs were simultaneously available. Detailed description of the set-up and procedure used for this experiment has been reported elsewhere (Shibata et al., 2014). Briefly, we used a 65-mm wide custom-made sensorized grip handle (two ATI F/T sensors) to measure grip and load digit forces and the three-dimensional coordinates of the center of pressure (CoP) of the thumb and index finger pad (Figure 1, Experiment 1). The handle was secured to the table to allow subjects applying normal and tangential digit forces without rotating or lifting the object. Subjects sat on a chair with both forearms resting on adjustable supports with the hand semi-pronated. At the beginning of each trial, an experimenter manually placed the thumb and index fingertip of the subject's right hand at the same vertical, collinear, starting position onto the sensorized handle. During this passive digit positioning, subjects were asked to relax their hand. Collinear fingertip position was defined as the vertical distance between thumb and index fingertip CoP (*d*_*y*_) equal to 0 ± 3 mm (i.e., both fingertips contact the handle at approximately the same height from the base of the device; Figure 1). Subjects had no visual feedback of the right hand. Once the digits were placed in collinear position, subjects were instructed to exert digit forces against the handle in one of six possible combinations of direction and magnitude: (1) both digits' tangential forces directed upward (*T*_*UP*_*-I*_*UP*_); (2) both digits' tangential forces directed downward (*T*_*DN*_*-I*_*DN*_); (3) thumb and index tangential forces directed upward and downward, respectively (*T*_*UP*_*-I*_*DN*_); (4) thumb and index tangential forces directed downward and upward, respectively (*T*_*DN*_*-I*_*UP*_); (5) no digit forces (“Control”, *Null*); and (6) only digit normal forces (*F*_*n*_ *only*). In all force combinations with the exception of *Null*, subjects were required to exert normal digit force between 4 and 5 N and vertical forces between ±2.5 and ±3.5N (positive and negative signs denote upward or downward direction, respectively). During the sense distance phase, subjects were asked to memorize the perceived *d*_*y*_ while complying with the instructed force combination during a 5-s period. Subjects were then asked to relax their hand on the table for 5 s, at the end of which subjects had to match the previously-experienced fingertip distance using the same hand within a 10-s period. Finally, once subjects reported having reached the matched fingertip distance, we asked them to hold the digit configuration for 5 s to record the matched *d*_*y*_. Thus, during the sense distance phase of Experiment 1 both tactile and non-tactile digit inputs were available to estimate the relative digit position of the thumb and index finger.

#### Experiment 2

This experiment was designed to quantify the individual contribution of tactile inputs for finger distance estimation. By means of two wearable haptic devices (Figure S1A) we delivered precise and repeatable tactile stimulations on subjects' thumb and index finger pads (Figure 1). The haptic devices generated normal and shear (tangential) forces by providing both compression and skin stretch on the fingertips, respectively. These stimulations aimed to reproduce the force-related tactile signals resulting from precision grips involved in Experiment 1. The range and magnitude of normal and tangential forces were the same required in Experiment 1. The three-dimensional coordinates of each digit CoP was calculated through an active motion capture system (Figure S1B). Unlike Experiment 1, subjects were not required to exert digit forces on a handle, but rather to memorize the perceived fingertip distance while experiencing tactile stimulation on the finger-pad. At each trial, subjects sit on a chair with the forearm and wrist stabilized by straps and rigid dowels anchored to a platform to minimize changes in posture across trials. Subjects also wore opaque goggles throughout the experiment to remove visual feedback of digit position and haptic devices. After device calibration (see Supplementary Material text for details), subjects' thumb and index fingertip attached to the device were passively moved by the experimenter to attain *d*_*y*_ equal to 0 ± 3 mm (i.e., collinear position) and CoP horizontal distance (*d*_*z*_) of 65 ± 3 mm. Once the target *d*_*y*_ and *d*_*z*_ were reached, the haptic devices were activated to apply normal and/or tangential forces (i.e., compression and skin stretch) on the finger pads at one of the above-described six force direction and magnitude combinations (see *Experiment 1* above). Under tactile stimulation, subjects sensed and memorized *d*_*y*_ for 5 s. Throughout the 5-s constant stimulation (Figure S2A), the experimenter supported the weight of the device with the volar aspects of his hands, to maintain *d*_*y*_and *d*_*z*_ within a tolerance window of ±3 mm. After tactile stimulation was gradually reduced to zero, the subject's hand was passively moved by the experimenter to rest on the supporting surface. After 5-s relax phase, subjects were required to actively move their digits to reproduce (i.e., match), within 10 s, the *d*_*y*_ they had experienced during tactile stimulation. Subjects maintained the matched *d*_*y*_ for 5 s, at the end of which they rested their hand and prepared for the following trial. Due to the lack of voluntary force production during sense distance in Experiment 2 subjects could rely only on tactile signals to estimate and memorize digit relative position.

#### Experiment 3

This experiment was designed to quantify the individual contribution of non-tactile inputs for finger distance estimation. The endpoints of two haptic devices (Phantom premium 1.5; Geomagic) were attached to the intermediate phalanx of the index finger and proximal phalange of the thumb through a rigid thimble and Velcro straps (Figure 1). Together, the Phantom thimble and the Velcro straps worked like a semi-rigid attachment ring that uniformly compressed the area of force application avoiding skin stretch in the direction of the force. The three-dimensional position of the endpoints attached to each digit (i.e., point of force application, PFA) was recorded by the devices' internal encoders and used to define vertical and horizontal position coordinates (i.e., *y* and *z* axis) of thumb and index finger. In accordance with Experiments 1 and 2 subjects were required to keep the vertical distance between digits PFA equal to zero (i.e., collinear position) and PFAs horizontal distance of 65 ± 3 mm. Phantom applied 2.9 N vertical forces and 2.3 N normal force on each digit PFA. During the Phantom force production phase, a sound was played when subjects' horizontal or vertical distance deviated ±3 mm from the nominal values. Each trial was composed of the same phases described for Experiments 1 and 2. Subjects sat on a chair with the forearm resting on a foam cushion and their hand relaxed. Subjects' forearm was stabilized by rigid dowels anchored to a platform to fix the position of the forearm and wrist relative to the haptic device throughout the experiment. Vision of right forearm, hand, and Phantom was occluded by a screen. At the beginning of each trial, subjects were asked to actively place the fingertips in collinear vertical position and 65 mm apart. A visual feedback composed of a target and cursor spheres were displayed on a monitor to provide the subjects with implicit information on the actual vertical and horizontal digit position. Subjects moved their digits such that cursor and target spheres position coincide to achieve collinear position (*d*_*y*_ = 0 mm). Once *d*_*y*_ and *d*_*z*_ values were reached and maintained in collinear position for at least 0.5 s visual feedback was extinguished and Phantom started to exert one of the above-described six force combinations (see *Experiment 1* above) on subjects' digits PFA (Figure S2B). Throughout force presentations, subjects were asked to exert digit forces against the Phantom endpoints forces without deviating from the initial *d*_*y*_ and *d*_*z*_ beyond the ±3 mm range. Following force exertion and digit position memorization, subjects were required to place the fingers in starting position and relax their hands for 5 s. Then, subjects reproduced the memorized finger position. Once subjects reported to have reached the previously-sensed configuration they kept digit positions for 5 s before returning to the start position and preparing for the next trial. Due to the lack of force related tactile signals on finger-pad in Experiment 3 subjects could rely only on non-tactile inputs, i.e., efference copy and proprioception, to estimate and memorize digit position during the sense phase.

Each digit force combination was randomly presented 6 times in Experiment 3 and 5 times in Experiments 1 and 2. Average duration of each experiment was approximately 1 h. Subjects were given rests every 10 trials or as requested by the subject to prevent digits muscles fatigue. Before Experiments 2 and 3 subjects underwent familiarization with the devices and task composed of 2 repetitions of each force combination.

### Data Processing and Testing Methods

For all experiments, we verified offline whether normal and tangential forces during the “*Sense distance”* phase were within the force limits imposed by the protocol. Force values from each experiment were submitted to two nonparametric analysis (data pooled across subjects, *n* = 50) to test that both normal and tangential forces were not statistically different than force thresholds. For Experiments 1 and 2 the *d*_*y*_ and *d*_*z*_ values were computed using the digit *y* and *z* CoP coordinates, respectively. For Experiment 3 the points of force application (i.e., PFA) of the Phantom endpoints were used for the analysis. For all experiments, *d*_*y*_ was defined as the vertical coordinate of thumb (CoP or PFA) minus the vertical coordinate of index finger (CoP or PFA), where positive and negative *d*_*y*_ denote the thumb as being higher or lower than the index fingertip, respectively. The *d*_*z*_ was defined as the horizontal distance between thumb and index finger CoP or PFA. Wilcoxon nonparametric analysis on *d*_*y*_ and *d*_*z*_ values, pooled across subjects and grouped by force conditions (*n* = 50), were performed to check whether vertical and horizontal digit distance were kept within the threshold ranges during sensing phase. Matching *d*_*y*_ errors (bias) were defined as the *d*_*y*_ measured during the “*Hold*” phase minus the *d*_*y*_ observed during the “*Sense distance*” phase. To account for each subjects' idiosyncrasies in *d*_*y*_ estimation (e.g., fingers length, perceptual bias in absence of any force-related input), matching errors measured during the *Null*, control, condition was subtracted from the *d*_*y*_ matching error obtained within all other conditions. Note that the resultant matching error can be positive or negative, hence quantifying both the magnitude and direction of subjects' bias in reproducing vertical fingertip distance. Finally, matching error values across subjects were submitted to a one-tailed nonparametric test to determine matching errors significantly different than 0 (Wilcoxon Signed rank test). Occurrence and magnitude of *d*_*y*_ matching errors in Experiments 2 and 3 were interpreted as measures of the contribution of tactile and non-tactile inputs to *d*_*y*_ estimation, respectively. Profiles of relative and absolute digit finger positions from a representative subject during sensing (*T*_*UP*_*-I*_*DN*_ force combination) and matching, in all three experiments, are shown in Figure 2.

### Relative Contribution of Tactile and Non-tactile Inputs

We further quantified the role played by tactile and non-tactile inputs by assuming that fingertip distance estimation relies on a linear combination of these two sources of information. We chose this approach as the most parsimonious quantification of sensory inputs integration, as done by other authors proposing linear combination of estimates from cues (Young et al., 1993) and based on recent evidence suggesting linear combination of tactile and proprioceptive signals in the somatosensory cortex (Kim et al., 2015). Following this assumption, we expected that:

(1) $$\begin{array}{r} Dyer_{comb}=Dyer_{t}+Dyer_{nt} \end{array}$$

where *Dyer*_*t*_ and *Dyer*_*nt*_ are the *d*_*y*_ matching errors measured when either tactile or non-tactile inputs were available, respectively, and *Dyer*_*comb*_ is the *d*_*y*_ matching error measured when both signals were presented in Experiment 1. Note that, unlike the design used in Young et al. (1993), we did not manipulate the reliability of sensory cues for computing their weights. Instead, we calculated tactile (α_*t*_) and non-tactile (α_*nt*_) contributions to digit distance estimation by means of the following formulae:

(2) $$\begin{array}{r} {\alpha_{t}\;=Dyer}_{t}\;/\left(Dyer_{t}+Dyer_{nt}\right) \end{array}$$

(3) $$\begin{array}{r} {\alpha_{nt}\;=Dyer}_{nt}\;/\left(Dyer_{t}+Dyer_{nt}\right) \end{array}$$

Where α_*t*_ or α_*nt*_ equal to 1 indicate exclusive contribution of tactile or non-tactile signals, respectively, for bias of fingertip distance estimation. In addition, we performed a further analysis based on cues reliabilities by comparing the inverse of the variance of the errors obtained in Experiments 2 and 3 with the inverse of the variance obtained during Experiment 1. The outcome of this calculation was interpreted as in Oruç et al. (2003), that is either supporting or rejecting the hypothesis of linear summation of tactile and non-tactile inputs underlying finger distance estimation.

### Strength of Bias Elicited by Tactile and Non-tactile Inputs

In addition to the individual contribution of tactile and non-tactile force-related signals to the *d*_*y*_ matching bias, we quantified the probability of each input to significantly affect sensing and memorization of digit relative position. We will refer to this measure as *strength of bias*. For each force combination condition, we calculated the probabilities of *d*_*y*_ matching error to be higher and lower than the third and first quartile of the *d*_*y*_ error distribution, respectively, observed during the *Null* condition. Thus, this analysis takes into consideration only the *d*_*y*_ errors that exceeded the range of errors (i.e., interquartile range) found in the control condition. In fact, the strength of bias quantifies the probability of tactile and non-tactile signals to elicit consistent rather than transient directional errors in finger distance estimation. To account for individual differences in the strength to bias, for each subject we subtracted the resulting probability of bias during the control condition (i.e., *Null* condition, no force related signals on the digits) from the probabilities extracted in all others force combinations. Since this measure can be expressed by probabilities of both positive and negative errors in the same condition, we considered the residual probabilities obtained by subtracting positive from negative *d*_*y*_ matching errors. Thus, strength of bias values equal to zero denote a similar tendency to produce opposite matching errors for a given force combination. Conversely, non-zero strength of bias indicates a consistent tendency to elicit matching error in a specific direction associated to a force combination.

As further quantification of the strength of the effect observed across experiments, we calculated the Cohen's *r* value associated to any statistical significant result. We calculated the effect size *r* in accordance with Pallant (2005), who suggested to extract the effect size from a one-sample non-parametric test by dividing the output of the test statistics by the square root of the number of observations.

## Results

We quantified subjects' ability to reproduce fingertip sensed and memorized vertical distance while normal and/or tangential forces were exerted with the thumb and index fingertips (Experiment 1), were experienced on the finger pads by means of wearable haptic devices (Experiment 2), or applied by the digits against a haptic device without experiencing force related signals on the finger pads (Experiment 3). In each experiment, we analyzed the difference between fingertip vertical distance (*d*_*y*_) observed in the sensing and matching phases, i.e., *d*_*y*_ matching errors. The presence of matching errors statistically different than zero across the experiments was interpreted as evidence of the contribution of tactile and non-tactile signals for the estimation of fingertip relative position.

### Inputs of Forces and Position During Sensing Phase

Statistical analysis confirmed that subjects complied to the task requirements during the sensing phase. Specifically, in all experiments *d*_*y*_'s were not significantly different from 0 and remained within the 0 ± 3-mm range (Figure S3A). Similarly, in Experiments 2 and 3 fingertip horizontal distance (*d*_*z*_) did not significantly exceed the 65 ± 3-mm range (Figure S3B). Tangential forces exerted by the digits (Experiments 1 and 3) or applied to the finger pads (Experiment 2) during the “*Sense distance*” phase were always within the target range (one-tailed Wilcoxon tests, *p* > 0.05; Figure S4A). Similar results were obtained by the analysis of normal forces for all the experiments (*p* > 0.05; Figure S4B). Overall, subjects exerted/sensed tangential and normal forces within the same range in all three experiments while sensing and memorizing digit collinear position.

### Matching Errors

**Figure 3:**
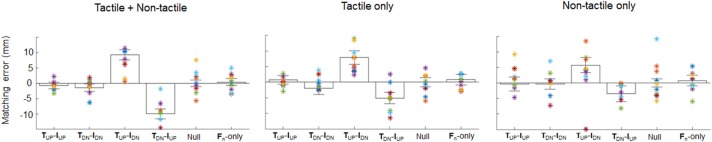
Matching error. Median and 95% confidence intervals (CI) of matching errors of Experiment 1 (left), Experiment 2 (center) and Experiment 3 (right) for each digit force combination. Asterisks denote the mean matching error averaged across the 5 repetitions of each subject in each condition. Positive errors denote higher position of thumb relative to index fingertip, and vice versa for negative errors. *T*_*UP*_*-I*_*UP*_: both digits' tangential forces directed upward; *T*_*DN*_*-I*_*DN*_: both digits' tangential forces directed downward; *T*_*UP*_*-I*_*DN*_: thumb and index tangential forces directed upward and downward, respectively; *T*_*DN*_*-I*_*UP*_: thumb and index tangential forces directed downward and upward, respectively; *Null*: no digit forces; and *F*_*n*_ *only*: only digit normal forces.

*d*_*y*_ matching errors obtained from all conditions of each experiment were modulated by both the direction of digit tangential forces (main effect of *Force condition*) and the available sensory inputs (main effect of *Experiment*). In all three scenarios, subjects accurately matched *d*_*y*_ when the direction of thumb and index finger tangential forces was the same (*T*_*UP*_*-I*_*UP*_, *T*_*DN*_*-I*_*DN*_), with matching error not being statistically different than zero (*p* > 0.05; Figure 3 and Table 1). Importantly, a Kruskal-Wallis non parametric ANOVA performed on the error exhibited in the three experiments during control (*Null* condition) and *Fn-only* conditions did not reveal any statistical differences across groups ($\chi_{149}^{2}$ = 0.02, *p* = 0.99 and $\chi_{149}^{2}$ = 0.24, *p* = 0.88 for *Null* and *Fn-only* conditions, respectively). Between groups similarity in these conditions can also be observed from Figure 3 showing individual performance. Therefore, the outcome of this analysis revealed that the three groups of subjects, in conditions of no lateral force input, estimated similarly finger distance. In contrast, when the directions of thumb and index finger tangential forces were opposite (i.e., *T*_*UP*_*-I*_*DN*_, *T*_*DN*_*-I*_*UP*_), subjects exhibited *d*_*y*_ matching errors that were statistically different than zero for all experiments (Figure 3 and Table 1). In particular, during condition *T*_*UP*_*-I*_*DN*_ the matching error median value exhibited in Experiments 1, 2, and 3 were respectively 9.2 mm (CI: 7.6, 10.9; *z* = 6.02, *p* < 0.01, effect size *r* = 0.81), 7.9 mm (5.7, 10.1; *z* = 4.52, *p* < 0.01, effect size *r* = 0.61) and 5.7 mm (3.3, 8.2; *z* = 3.47, *p* < 0.01, effect size *r* = 0.47). Similar trend of *d*_*y*_ matching error, but with opposite sign, were observed for condition *T*_*DN*_*-I*_*UP*_: Experiment 1, −9.9 mm (CI: −11.6, −8.3; *z* = −6.38, *p* < 0.01, effect size *r* = 0.86), Experiment 2, −5.1 mm (−6.9, −3.3; *z* = −3.84, *p* < 0.01, effect size *r* = 0.52) and Experiment 3, −3.5 mm (−6.0, −1.0; *z* = −3.35, *p* < 0.01, effect size *r* = 0.45). The magnitude of *d*_*y*_ matching errors associated with opposite conditions decreased when tactile and non-tactile signals were presented individually (~3.5 and ~6.4 mm for the *T*_*UP*_*-I*_*DN*_ and *T*_*DN*_*-I*_*UP*_conditions, respectively, across experiments). Two Kruskal-Wallis no parametric tests with experiment as between-group factor were performed separately on the matching errors obtained in *T*_*UP*_*-I*_*DN*_ and *T*_*DN*_*-I*_*UP*_ conditions. This analysis revealed a statistically significant effect of experiment for *T*_*UP*_*-I*_*DN*_ and *T*_*DN*_*-I*_*UP*_ ($\chi_{2,\;164}^{2}$ = 8.89, *p* = 0.01, and $\chi_{2,\;164}^{2}$ = 19.8, *p* < 0.01, respectively). *Post-hoc* tests on data from the *T*_*UP*_*-I*_*DN*_ condition revealed no significant differences between matching errors measured when both tactile and non-tactile inputs in Experiment 1 were available vs. tactile signals only in Experiment 2 (*p* = 0.22). In contrast, we observed a significant reduction in matching error when only non-tactile inputs were available in Experiment 3 compared to when both tactile and non-tactile inputs were available in Experiment 1 (*p* = 0.01). In the *T*_*DN*_*-I*_*UP*_ condition, *post-hoc* tests revealed a significant decrease of the matching error associated to isolated (i.e., Experiment 2 and 3) vs. combined tactile and non-tactile inputs (i.e., in Experiment 1; both *p* < 0.01).

**Table 1:** Median matching errors and confidence intervals.

	**T_**UP**_-I_**UP**_**	**T_**DN**_-I_**DN**_**	**T_**UP**_-I_**DN**_**	**T_**DN**_-I_**UP**_**	**Null**	**F_**n**_-only**
**Exp. 1**	−1.77 ≤ 0.7 ≤ 0.35	−2.63 ≤ −1.4 ≤ 0.22	**7.59 ≤ 9.2 ≤ 10.92**	**−11.59 ≤−9.9 ≤−8.33**	−1.06 ≤ 0.0 ≤ 1.06	−0.75 ≤ 0.4 ≤ 1.51
**Exp. 2**	−0.69 ≤ 0.7 ≤ 2.18	−3.98 ≤ −1.96 ≤ 0.05	**5.72 ≤ 7.9 ≤ 10.09**	**−6.90 ≤−5.1 ≤−3.28**	−1.45 ≤ 0.0 ≤ 1.49	−0.87 ≤ −0.8 ≤ 2.53
**Exp. 3**	−2.54 ≤ −0.3 ≤ 1.95	−1.98 ≤ −0.35 ≤ 1.28	**3.29 ≤ 5.7 ≤ 8.19**	**−5.99 ≤−3.5 ≤−0.94**	−1.33 ≤ 0.0 ≤ 1.33	−0.96 ≤ 0.8 ≤ 2.5

*Lower, median and upper 95% distribution values of d_y_ matching error (mm) for each experimental scenario (data pooled across all subjects and trials, n = 50). Bold text denotes matching error values that are significantly different than 0 (Wilcoxon signed rank test, p < 0.01)*.

### Correlation Between Muscle Fatigue and Matching Errors

**Figure 4:**
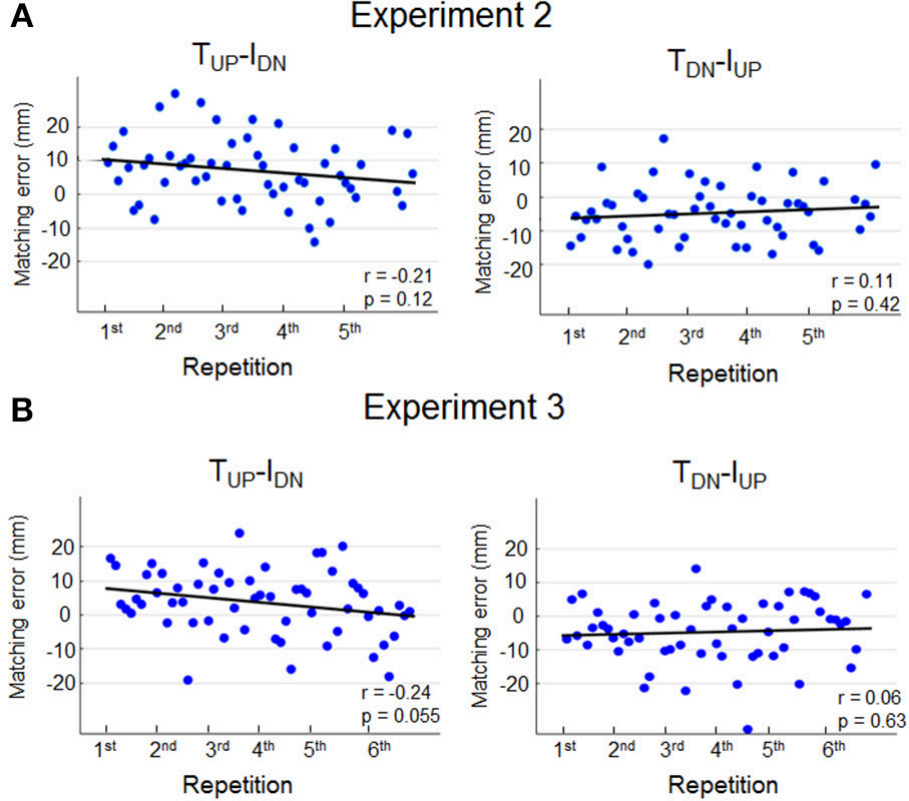
Matching errors as function of repetition. Single trial matching error exhibited by each subject during *T*_*UP*_*-I*_*DN*_ and *T*_*DN*_*-I*_*UP*_ is plotted against repetitions in Experiment 2 **(A)** and Experiment 3 **(B)**. Data from all subjects are shown for each condition and experiment, and ordered as a function of repetition, e.g., the first 10 dots depicted in the left plot (Experiment 2A) denote the first repetition of T_UP_-I_DN_ force combination of each one of the 10 subjects. Pearson correlation coefficient (*r)* and regression statistics (*p)* is reported in each plot.

In light of previous evidence demonstrating that muscle fatigue affects limb position estimation (Carpenter et al., 1998; Allen et al., 2010; Vafadar et al., 2012) we performed further analysis to determine whether, in the current study, potential finger muscle fatigue caused by wearing the device on the finger pad (Experiment 2) might have contributed to overestimate *d*_*y*_. If muscle fatigue were influencing sensing and memorization of finger position, perceptual bias should have increased with the number of trials. Figure 4A shows that for any of the two opposite force conditions subjects exhibited no significant correlations between matching error magnitude and number of trials.

History of muscle contraction (thixotropy) has also been shown to affect perception of limb position (Proske and Gandevia, 2012; Proske et al., 2014). Figure 4B shows the result of additional analysis performed on the data of Experiment 3 to determine whether thixotropy might have contributed to the bias in *d*_*y*_ estimation. Similarly to Experiment 2, matching errors did not significantly change as function of number of trials, hence suggesting that subjects' sensing and memorization were not influenced by the history of muscle contractions.

### Contribution of Tactile and Non-tactile Inputs to the Estimation of Fingertip Vertical Distance

**Figure 5:**
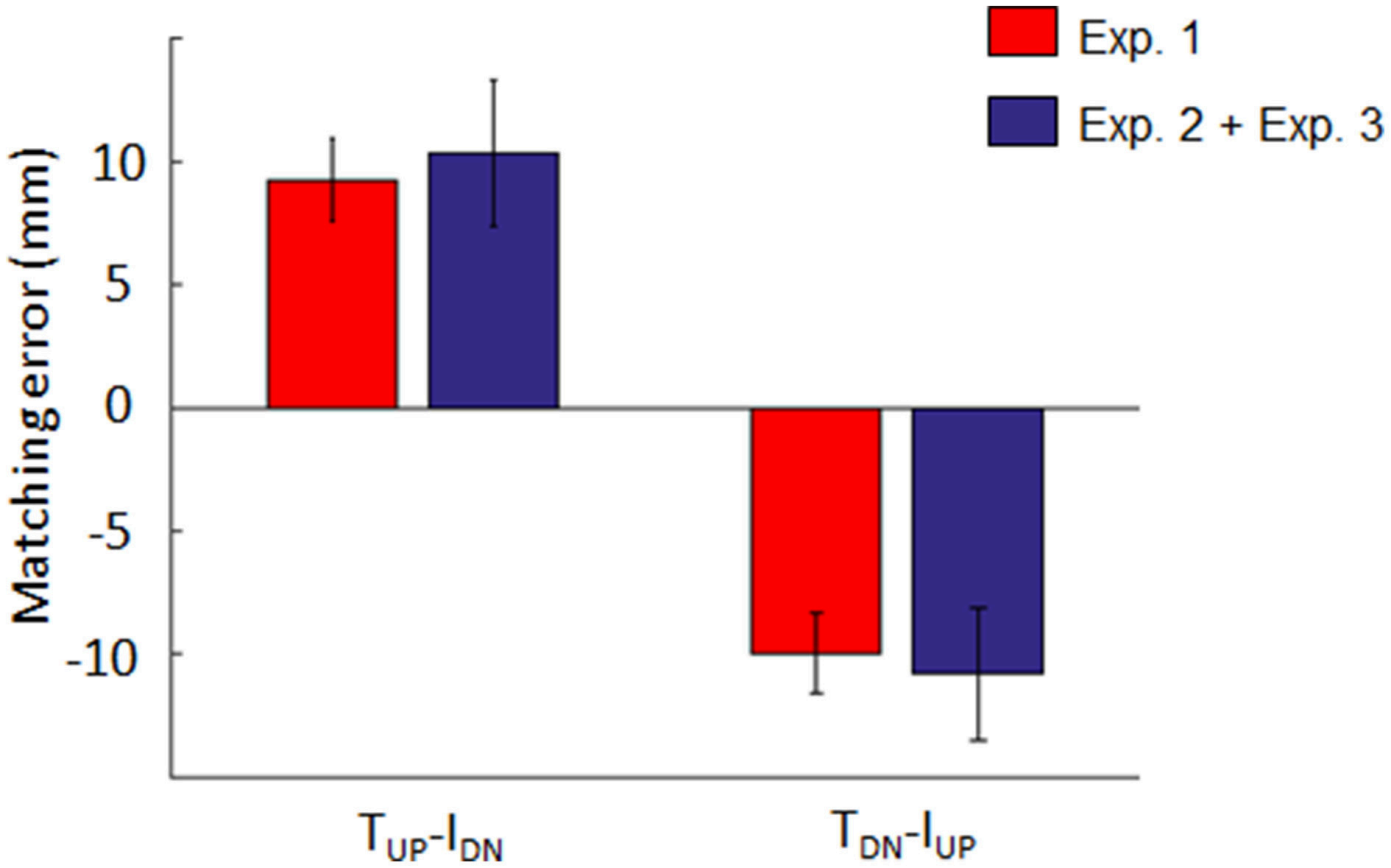
Individual and combined bias elicited by tactile and/or non-tactile inputs. Blue bar: median ± 95% CI of the sum of matching errors exhibited in Experiment 2 (tactile inputs only) and 3 (non-tactile inputs only). Red bar: median ± CI matching error observed when both inputs were simultaneously presented (Experiment 1).

Figure 5 shows the results of the analysis performed to test the hypothesis that *d*_*y*_ estimation depends on a linear combination of tactile and non-tactile inputs. For both opposite conditions (*T*_*UP*_*-I*_*DN*_ and *T*_*DN*_*-I*_*UP*_), the distribution of the sum of the individual contributions of tactile and non-tactile inputs recorded during Experiment 2 and 3 (blue bars, respectively) approximated the distribution of bias found when these inputs were simultaneously available (Experiment 1; red bars in Figure 5). For the *T*_*UP*_*-I*_*DN*_ condition, the median ± CI of the sum of the biases in Experiment 2 and 3 was 10.3 mm (7.4, 13.3). Similarly, for the *T*_*DN*_*-I*_*UP*_ condition, the combined bias was −10.8 mm (−13.5, −8.1). Two no parametric tests performed on the *T*_*UP*_*-I*_*DN*_ and *T*_*DN*_*-I*_*UP*_ matching error distributions revealed no significant difference between the bias obtained when both tactile and non-tactile information were available and the sum of the biases elicited by either type of input (*z* = 0.76; *p* = 0.44 and *z* = −0.09; *p* = 0.92 for *T*_*UP*_*-I*_*DN*_ and *T*_*DN*_*-I*_*UP*_, respectively). Similar results were obtained in all other conditions where lateral force was provided, i.e., *T*_*UP*_*-I*_*UP*_ (*z* = −0.03; *p* = 0.97), *T*_*DN*_*-I*_*DN*_ (*z* = −0.25; *p* = 0.8). During opposite force conditions, tactile inputs contribution was 0.58 and 0.60 for *T*_*UP*_*-I*_*DN*_ and *T*_*DN*_*-I*_*UP*_ conditions, respectively, hence tactile signals always providing a greater contribution to the bias than non-tactile inputs.

**Figure 6:**
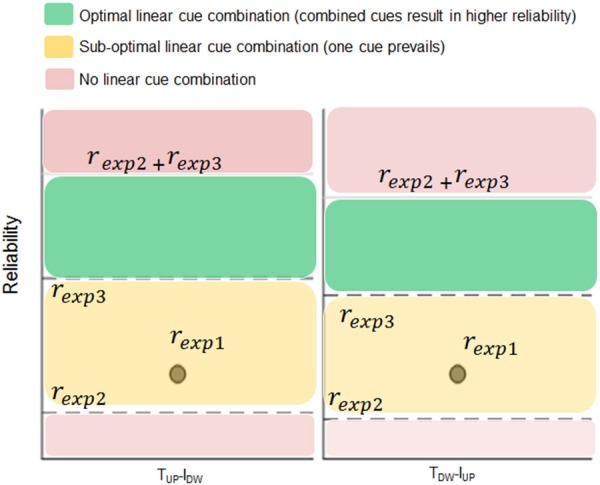
Reliability based cue combination analysis. Inverse of the variance (i.e., reliability) of the matching errors exhibited in experiment 1 (*r*_exp1_; dot), 2 (*r*_exp2;_ lower dashed horizontal line), and 3 (*r*_exp3_; upper dashed horizontal line) for condition *T*_*UP*_*-I*_*DN*_and *T*_*DN*_*-I*_*UP*._Position of *r*_exp1_ within the plot supports (green and yellow areas) or reject (pink area) the hypothesis of linear combination of tactile and non-tactile signals for finger distance estimation.

The outcome of the analysis based on cues reliability is shown in Figure 6. As it can be noticed, in both the experimental conditions where we observed a statistical significant bias (*T*_*UP*_*-I*_*DN*_and *T*_*DN*_*-I*_*UP*_) the reliability exhibited in Experiment 1 (*r*_exp1_) is higher than the reliability of the Experiment 2 (*r*_exp2_) but lower than the reliability observed in Experiment 3 (*r*_exp3_). This result indicates that when both tactile and non-tactile inputs of force were provided simultaneously, the linear summation occurred (*r*_exp1_is higher than *r*_exp2_), yet it was not optimal, namely the reliability of the estimate based on both cues, i.e., *r*_exp1_, resulted lower than the reliability of the estimate based on non-tactile cue (*r*_exp3_).

### Strength of Bias

**Figure 7:**
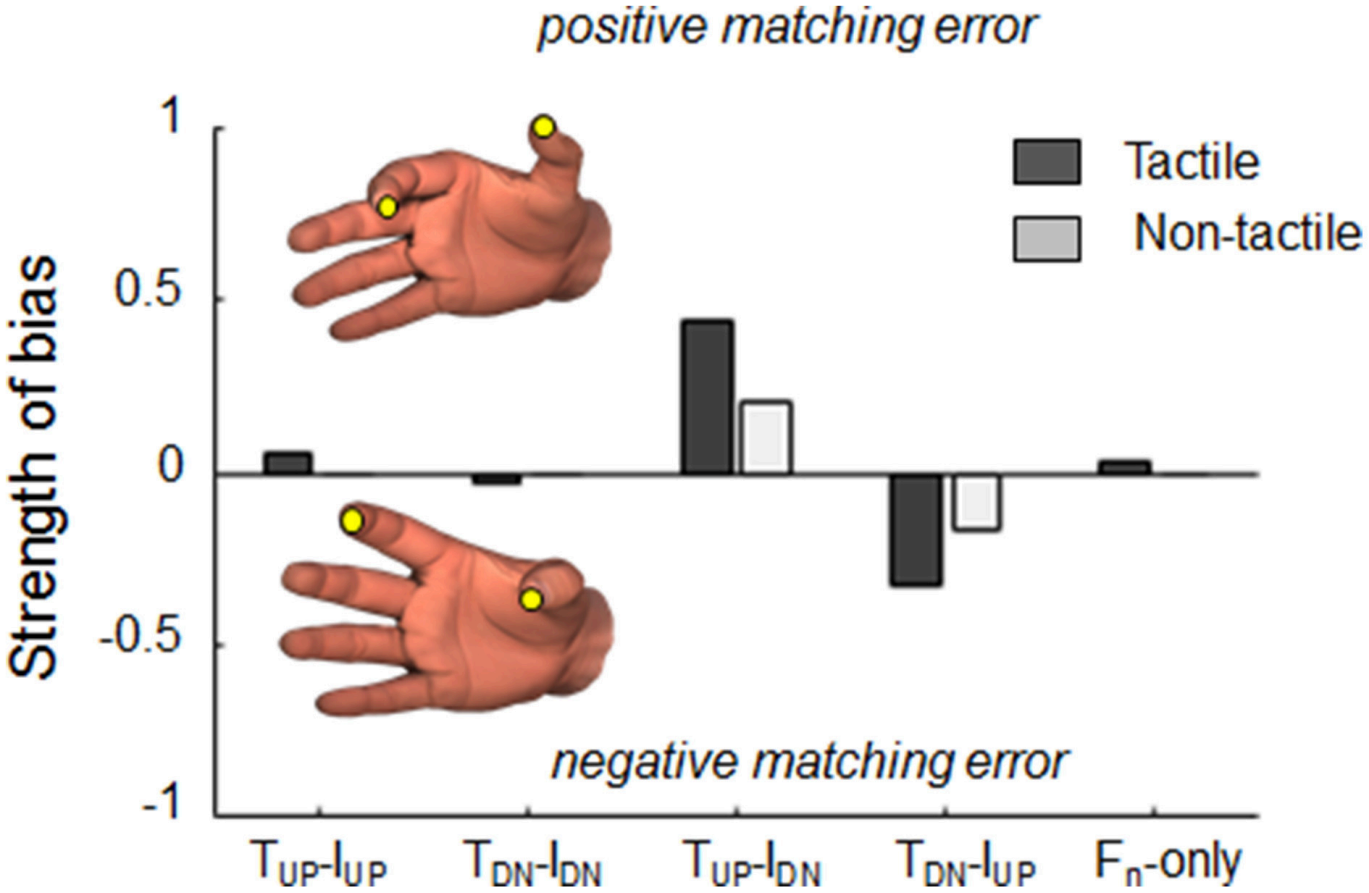
Strength of bias. Probability to elicit positive or negative consistent *d*_*y*_ matching errors, i.e., thumb higher (positive) or lower (negative) than index fingertip, respectively, when either tactile (dark bars) or non-tactile (light bars) force related signals were available. For each force condition, probabilities were calculated by testing whether the error exhibited in each of the 50 trials was within the inter-quantile range of errors exhibited during baseline condition (null).

Our analysis of the strength of bias revealed that when tangential forces were either actively exerted or passively experienced in the same direction, subjects' estimation were characterized by small (<5%) probabilities of consistent bias (Figure 7). In contrast, tangential force combinations characterized by opposite directions revealed different probability of eliciting consistent matching errors when either tactile and non-tactile inputs were available. When subjects could rely only on force-related tactile inputs to memorize digit position, probabilities of bias doubled (44 and 33% for positive and negative errors during *T*_*UP*_*-I*_*DN*_ and *T*_*DN*_*-I*_*UP*_, respectively; dark gray bars) with respect to when only non-tactile inputs were available (20 and 17%, respectively; light gray bars).

## Discussion

In the current study we assessed the presence and strength of bias in fingertip distance estimation associated to tactile and non-tactile force-related inputs experienced in isolation. We report three main findings: (a) tactile and non-tactile inputs alone contribute to the estimation of fingertip distance; (b) tactile inputs associated to finger pad deformation contributed the most to the estimation of fingertip relative position and were more likely to elicit consistent perceptual bias; (c) tactile and non-tactile signals appear to be integrated in a linear fashion.

### Contribution of Tactile and Non-tactile Signals for the Estimation of Fingertip Relative Position

Our results show that tactile and non-tactile signals experienced in isolation contribute to the estimation of fingertip vertical distance. By stimulating finger pad mechanoreceptors through custom-designed wearable haptic devices in different direction combinations, we provide novel evidence that tactile signals are used to build a representation of fingertip relative position. This result is consistent with previous studies demonstrating that ensembles of cutaneous receptors can encode magnitude and direction of forces acting on the finger pad (Birznieks et al., 2001; Panarese and Edin, 2011). Our findings are also consistent with recent evidence that stimulation of cutaneous mechanoreceptors caused by finger pad deformation and skin stretch on the dorsal side of the hand can induce misperception of finger position and displacement (Edin and Abbs, 1991). For example, tactile afferents stimulated by changes in the contact area have been shown to provide proprioceptive cues about finger displacement (Moscatelli et al., 2016). Moreover, it has been recently suggested that tactile inputs contribute to the perception of direction of upper limb motion as a result of the integration of these signals with motor related proprioceptive inputs (Bianchi et al., 2017). While these previous investigations focused on sensorimotor mechanisms underlying estimation of individual digit position and displacement, our results extend their findings by revealing that tactile signals also contribute to a higher-level representation of digit positions involving more complex sensorimotor transformations, i.e., estimation of fingers distance. Taken together, previous and our work suggest that tactile signals play a greater role for the estimation of relative fingertip position (~60%) rather than the perception of limb motion direction (~17% from Bianchi et al., 2017). Although our approach allowed us to quantify the relative contribution of each input in isolation with respect to when they were concurrently available (Figure 3), two main limitations of our design might be pointed out as potentially influencing our findings. First, it might be argued that the present study considered a relatively small number of subjects. While a wider sample might have enhanced the size of the effect we have observed, in the current study we compensated to the limitation of a relatively small number of subjects, by performing non-parametric statistics on the data pooled across subjects and repetitions (*n* = 50). In doing so, the sample considered accounted for the whole (within and between) variability of each group. Importantly, the employment of non-parametric statistics ensured the reliability our results, being such a statistics applicable also on relatively small sample size and not greatly affected by outliers (Nham, 2016). The second limitation is associated to the between group design employed in the present study. Since, the group of subjects tested in each experiment were different, it might be hypothesized that the differences observed across experiments are due to group idiosyncrasies in estimating finger distance, rather than different tactile and non-tactile signals contributions. With this regard, it should be noted that each subjects group was randomly sampled, hence if any between-groups differences were affecting our results, they were randomly distributed across the three experiments. Additionally, within and between group differences in estimating finger relative position were compensated by subtracting the mean of error exhibited during control condition (Null force) from the error exhibited in all the other conditions. In fact, in condition of no tangential force signals, the three groups exhibited no statistical differences in finger distance estimation, hence supporting the assumption that they were equally sensitive in estimating finger distance. Finally, the significant matching error observed in each experiment was characterized by a robust effect size (*r* > 0.4), which suggests that the main differences exhibited across experiments cannot be attributed uniquely to between groups differences.

### Tactile Inputs Contribute the Most to Perception of Digit Relative Position

Our results revealed that when both force-related inputs are combined, tactile afferents play a primary role for the estimation of fingertip distance (Figure 7). Previous work has provided evidence for greater reliance on tactile inputs over proprioceptive and motor-related signals, whereby tactile cues override non-tactile input coding for illusory finger and hand movements (Robles-De-La-Torre and Hayward, 2001; Blanchard et al., 2011; Moscatelli et al., 2016; Bianchi et al., 2017). We propose two non-mutually exclusive explanations for the greater contribution exhibited by tactile inputs for digit distance estimation. First, information provided by non-tactile inputs may be intrinsically less reliable than that provided by tactile inputs, given the non-linear mapping between hand muscles and force production, and the different geometry of thumb and index finger joints. Second, when two functionally-related hand muscles are simultaneously contracted, the CNS is unable to accurately disambiguate the targets of two distinct motor commands (Kilbreath and Gandevia, 1994). While this limitation would not be detrimental in grasp control context characterized by low level sensorimotor adjustments, it might penalize digits position estimation tasks where the CNS has to integrate non-tactile inputs from muscles acting to generate net opposite fingertip forces.

### Linear Combination of Tactile and Non-tactile Inputs of Digit Forces Drives Estimation of Fingers Relative Position

The results of Experiments 2 and 3 indicate that neither tactile nor non-tactile inputs alone can account for the bias observed when both signals were available (Experiment 1, Figure 3). Our analysis based on cues reliabilities as well as our finding that the sum of matching error of the two experiments approximated the perceptual bias exhibited in Experiment 1 suggests that fingertip distance estimation occurs through a linear combination of digit-force related tactile and non-tactile inputs. Importantly, we found that combination of these signals is dynamic, namely that their integration is modulated with respect to the characteristic of the inputs associated with each digit, i.e., same vs. opposite force directions. In fact, tactile and non-tactile induced bias was greater when vertical force signals were applied or exerted in opposite directions (*T*_*UP*_*-I*_*DN*_ and *T*_*DN*_*-I*_*UP*_; Figure 7). Interestingly, when no vertical forces were exerted or experienced by the subjects (*F*_*n*_*-only*), only tactile signals are associated to a consistent bias, though very small (Figure 7). We interpret these findings as evidence that when the same force-related signals are provided to both finger pads, the estimation of finger relative position might be mainly driven by tactile signals. Conversely, in contexts where perception of fingertip distance relies on opposite tactile and non-tactile input, efference copy and proprioception would play a greater role in driving perception. Such integration may enhance the estimation of digit relative position based on opposite inputs of force. This framework is compatible with the hypothesis that sensory cues are integrated through a dynamic linear combination, changing the contribution of each input to the final percept in a context-dependent fashion (Young et al., 1993).

Our results are consistent with recent neurophysiological evidence obtained from neural recording of somatosensory cortex in non-human primates (Kim et al., 2015). In particular, the finding that neural representation of hand configuration is influenced by inputs from fingers' tactile afferents is consistent with our observation that bias in fingertip distance estimation is greater when tactile and non-tactile inputs are both available. Therefore, our results suggest that when multiple sources of force-related inputs are available, a representation of fingertip distance would depend on a linear combination of signals that is likely to occur, but is not limited to, the primary and secondary somatosensory cortex.

### Neural Representation of Fingertip Relative Position

**Figure 8:**
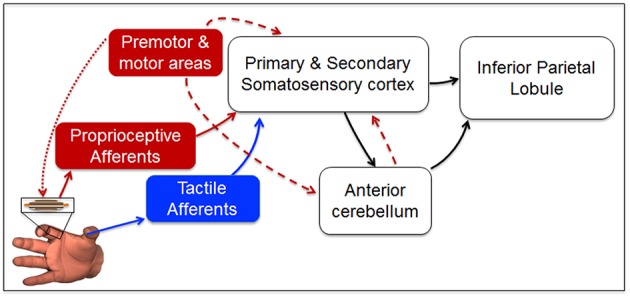
Putative sensorimotor network for fingertip distance estimation during manipulation. Direction and magnitude of force-related signals to/from the digits during manipulation are encoded by ensembles of tactile mechanoreceptors (blue arrow Birznieks et al., 2001; Johansson and Flanagan, 2009, and muscle spindle afferents (solid red arrow; Goodwin et al., 1972; Burke et al., 1988; Ribot-Ciscar et al., 2003; Smith et al., 2009; Dimitriou and Edin, 2010). Tactile and proprioceptive inputs reach primary somatosensory cortex (areas 3b and 3a, respectively; Thach, 1967; Jenmalm et al., 2003). Unimodal and multimodal neurons in primary and secondary somatosensory cortex integrate (Kim et al., 2015) and convey sensory information to the anterior lobe of cerebellum and the inferior parietal lobule (Clower et al., 2001). Here, further sensory integration occurs to enable body representation (Longo et al., 2010) and predictions about future body state (Hua and Houk, 1997; Blakemore and Sirigu, 2003; Pynn and De Souza, 2013). Motor cortex controlling digit force production (dotted red arrow) contributes to fingertip distance representation through an efference copy (dashed red arrow) sent to the somatosensory cortex (Blakemore et al., 1999) and the cerebellum (Hua and Houk, 1997), both projecting to parietal cortex (Clower et al., 2001). Blue box and blue arrows denote the putative network primarily involved in Experiment 2, whereas red boxes and red arrows denote the network that might have been involved in Experiment 3. White boxes and black arrows represent the neural network more likely to be involved in each of the three experiments when both (or either) tactile and non-tactile signals are available to estimate finger relative position (Kavounoudias et al., 2008).

Our findings provide insights on the putative sensorimotor brain network responsible for the transformation of force-related tactile and non-tactile inputs to a neural representation of fingertip relative position. The observation that tactile and non-tactile signals alone are necessary, but not sufficient, to account for perceptual bias found when both inputs are available, suggests that fingertip distance estimation relies on a higher-order representation of these signals. While ensembles of cutaneous and muscle spindle receptors have the remarkable ability to convey information about magnitude and direction of force-related stimuli to somatosensory cortex (Thach, 1967; Birznieks et al., 2001) they cannot—by themselves—account for the whole bias reported in our study. We propose that during dexterous manipulation, neural representation of fingertip relative position might emerge from the projections of signals conveyed to the somatosensory cortex to areas involved in building body representation, multisensory integration, and predictive motor control, such as the inferior parietal lobule, i.e., IPL, and the cerebellum (Figure 8). This framework is consistent with a large body of experimental evidence of a strong correlation between activity of somatosensory cortex and both inferior parietal lobule and the anterior lobe of cerebellum in the presence or absence of voluntary movements. Specifically, a previous study has identified a network involving these brain areas to be activated during unimodal and multimodal proprio-tactile integration for kinesthetic perception (Kavounoudias et al., 2008). Accordingly, our theoretical model is that, regardless of the sensory modality involved and the presence or absence of efference copy, the encoded direction of the force-related signals conveyed to the somatosensory cortex is projected to both cerebellum and IPL to compute predictions of sensory consequences (Hua and Houk, 1997; Blakemore et al., 1999; Clower et al., 2001; Pynn and De Souza, 2013) and representations of finger relative position (Longo et al., 2010).

## Conclusions

Estimation of where fingertips are relative to each other is a critical component of dexterous manipulation. While skin, joints and muscles receptors contribute to estimate the position of individual limb, the relationship between body parts (i.e., relative distance) is derived by higher level integration of somatosensory and motor signals (i.e., central body representation, Longo et al., 2010; Butler et al., 2015). Due to the lack of dedicated receptors encoding inter-limbs distance, perception of fingertip relative position relies on multiple sources of afferent and efferent signals, making it difficult to determine the relative role of each type of input. The present study provided evidence for a greater role of tactile signals and their linear integration with non-tactile inputs for estimation of fingertip distance.

## Data Availability Statement

Data set and main codes used for the data analysis are available at https://osf.io/a7g9p/, OSF repository.

## Author Contributions

ST, DS, and MS conceived and designed the research, interpreted the results, and drafted the manuscript. ST, DS, and FC set up the experimental apparatus. ST and DS performed the experiments, performed the data analysis, and prepared the figures. DP provided advices and support for the development of the customized haptic device. All authors edited and approved the final version of the manuscript.

### Conflict of Interest Statement

The authors declare that the research was conducted in the absence of any commercial or financial relationships that could be construed as a potential conflict of interest.
