# Supplementary material for: Linear Integration of Tactile and Non-tactile Inputs Mediates Estimation of Fingertip Relative Position
Source: Front Neurosci. 2019 Feb 11;13:68. doi: 10.3389/fnins.2019.00068 (PMC6378372; doi:10.3389/fnins.2019.00068)
Supplement: Supplementary file 1 [file Data_Sheet_1.PDF]

Supplementary Material

Linear integration of tactile and non-tactile inputs mediates estimation of fingertip relative position

Simone Toma,[1] Daisuke Shibata,[2] Francesco Chinello,[3,4] Domenico Prattichizzo,[3] Marco Santello[1*]

[1]School of Biological and Health Systems Engineering, Arizona State University, Tempe, Arizona, USA

[2]Athletic Training Education Program, Department of Health Exercise and Sports Science, University of New Mexico, Albuquerque, New Mexico, USA

[3]Department of information engineering, University of Siena, Siena, Italy

[4]Department of business development and Technology, Aarhus University, Midtjylland, Denmark

***Correspondence:**

Marco Santello
School of Biological and Health Systems Engineering
501 East Tyler mall, ECG Building, Suite 334
Arizona State University
Tempe, AZ 85287-9709
Ph.: +1-480-965-8279
Fax: +1-480-727-7624
E-mail: marco.santello@asu.edu

Supplementary Text

Methods

Apparatus

Experiment 2

The aim of this experiment was to reproduce the tactile signals associated to tangential and normal forces exerted by the thumb and index finger during precision grip in the absence of voluntary commands and proprioceptive inputs normally associated with force production.

Wearable haptic device. We displaced wearable haptic device platforms to stretch and compress the skin of the thumb and index finger pad using two servo motors (HS53 and HS5035HD) attached to two gear systems in each device (figure S1A). Motors' input and output were managed by a 3.0 Arduino board (Atmel/Atmega 328) communicating via USB with a PC. A force sensor (FSR400, short tail) placed on the device platform in contact with the finger pad was used to quantify the actual compressive force exerted on the fingertip. Skin stretch and compression resulted respectively from displacements parallel and orthogonal to the finger pad (*y- and z-* axis, respectively) of the movable platforms. The force sensors embedded in the device were read by an Arduino board, processed, and sent serially for storage to custom-written software (Matlab, The MathWorks, Natick, MA). The total mass of each haptic device was 45 g.

Since the output of the force sensors embedded in the device was in Volts (level of voltage), we map the sensor output in levels of force (Newton) by means of an ATI force/torque sensor. The scaling factor value obtained during these preliminary measures was then multiplied to the continuous output of the F/T sensors in the Arduino code to verify online that normal force produced by the device did not exceed the predefined force threshold (see main text for details on force ranges). Although we could not use a F/T sensor for the simulated shear force (F_{tg}) generated by the device, this force was estimated offline at the end of every trial as follows:

$$F_{tg} = L(\alpha^{rad}) \cdot F_n \cdot \mathrm{k} \qquad [1]$$

where *k* is a constant representing the elasticity of the skin (assumed to be linear), *L* quantifies the displacement of the device platform parallel to the fingertip (which is proportional to the rotational angle of the lateral stretch motor, α^{rad}), and F_n is the simulated normal force estimated by the FSR400 sensor. Each device could simulate up to 6 N·cm shear force applied to the finger pad. To maintain the shear forces within the target force range, preliminary tests were performed to identify the range of values of *L* and F_n that produced shear forces within the desired range.

Center of pressure calculation. CoP was calculated for each digit by creating a device local reference frame. This was defined based on the position of 4 active markers (Impulse; PhaseSpace, 8 cameras, frame rate: 480 Hz; spatial accuracy: ~1mm; spatial resolution: 0.1 mm) placed on the devices' extended bars (figure S1B). The local reference frame (*A*, centered at P_A, figure S1B) was compared with the global coordinate frame (*S*) of the motion capture system to compute the center of the platform (*P*) using the following transformation matrix:

$$P_s = \begin{bmatrix} R_S^A & d_{SA} \\ 0 & 1 \end{bmatrix} \cdot P_A \qquad [2]$$

where the array $A \in \mathbf{R}^4$ represents the position of the center of the local reference frame connected to the device, and $S \in \mathbf{R}^4$ is the center of the global reference frame S of the motion capture system. R_S^A and d_{SA} are the rotation and the distance between A and S, respectively, measured by the motion capture system. Thus, P_S denotes the coordinates of P with respect to S, starting from the position of P with respect to P_A. The digit CoP three-dimensional coordinates (P_S) resulting from this transformation was recorded and used for offline analysis.

Device calibration. We performed a calibration procedure at the beginning of every experimental session to compensate for inter-individual differences in finger pads compliance. During this procedure, the thumb and index finger pads were fixed to the device by Velcro straps and the compression force threshold was set to 1 N. Subsequently, the device was activated to compress each finger pad until the threshold value was read by the FSR400 sensor. Once the threshold was reached, the device decompressed the finger by increasing the distance between the device platforms of 1-mm. The resultant platform position was recorded and used as the starting position at the beginning of each trial. This calibration procedure ensured consistent forces on thumb and index within and across subjects. A new calibration was performed and the trial repeated if either the shear or compressive forces were above or below the target range.

Experiment 3

We designed this experiment to quantify the effects of normal and tangential force production on digit position estimation in absence of tactile inputs from the thumb and index finger pads.

Normal and tangential forces (Phantom *z*- or *y*-axis, respectively) were presented together or separately. When tangential and normal forces were presented together, the latter was measured as the force exerted by the digits against two virtual walls and rendered as a bi-directional virtual spring damper (0.25 N/mm spring constant and 0.02 N·s/mm damping constant). Since the sum of minimum tangential and normal forces used in Experiments 1 and 2 were larger than the total force range that each Phantom arm could exert (6.5 N > 6 N), subjects in this experiment were required to produce normal forces of ~2 to 2.5 N with each digit against the virtual walls. We considered this small difference in normal force across experiments (≈2 N, figure S4B Experiment 3) as irrelevant to subjects' matching performance based on previous evidence of no correlation between amount of exerted digit normal force and the magnitude of the bias on fingertip distance matching (Shibata et al., 2014).Throughout the experiment, movement of each digit was always constrained within a virtual plane parallel to the subjects' frontal plane located approximately 50 cm from subjects' chest. Visual feedback and written instructions were provided in a monitor placed approximately ~90 cm in front of the subject. The whole virtual reality (VR) environment was rendered through the CHAI3d library (Conti et al., 2005) included in a customized C++ code that stored time, force, and digit position data.

Data analysis

Experiment 1. Force and torque data were acquired, recorded, and stored in a computer with 12-bit A/D converter board through a custom data acquisition interface (LabVIEW version 8.0, National Instruments). During data collection, force data were

filtered online to compute and display digits CoP, normal and tangential forces. Data analysis was performed offline using custom-written software (Matlab, The MathWorks, Natick, MA). CoP values were used to calculate fingertip vertical distance (d_y) across trials, while normal and tangential force data were used to assess online possible deviations from the instructed range of forces.

Experiment 2. We computed digits CoPs offline after filtering position data from the markers with a low-pass Butterworth filter (5th order; cutoff frequency: 30 Hz). Normal and tangential force data were calculated as described in the main text (see *Apparatus,* main text).

Experiment 3. We computed d_y using the difference of the vertical coordinates of the two Phantom endpoints attached to subjects' right hand intermediate phalanx of the index finger and proximal phalange of the thumb. We estimated the tangential force exerted by subjects' finger (τ) by means of the following equation:

$$\tau_i = \mathrm{F} \cdot (1 - \sin(\alpha_i)) \qquad [3]$$

where *i* represents either the thumb or the index finger, F is the force exerted by the phantom arms against subjects' fingers at point of attachment (i.e., Point of Force Application), and α is the elevation angle calculated from the time-varying changes of each finger position (Phantom attachment) along the three coordinates (*cart2sph*, Matlab function). Specifically, when fingers counteracted Phantom force without moving (finger parallel to the ground, $\alpha = 0°$), the force applied by the finger was assumed to be the same as the force exerted by the Phantom (2.9 N). Conversely, when the subject's digit moved in the same or opposite direction of Phantom force (i.e., $\sin(\alpha_i) \neq 0$), the finger produced either a lower ($\sin(\alpha_i) < 0$) or higher ($\sin(\alpha_i) > 0$) force than the Phantom force.

Similarly, digit normal force exerted was extracted from the following formula:

$$\eta_i = \mathrm{F} \cdot (1 - \sin(\beta_i)) \qquad [4]$$

where *i* denotes thumb or index finger, F is the force exerted by the Phantom arms against subjects' digits (F_n*-only* condition) or the force exerted by the digits against the simulated virtual wall, and β is the azimuth angle calculated from the time-varying changes of each finger position (Phantom attachment) along the three coordinates.

In the main text force conditions labels characterizing Experiment 2 and 3 are defined with respect to the finger force direction experienced by the subjects, rather than the nominal tangential force direction exerted by the wearable haptic devices and the Phantom.

Profiles of digits positions during sensing and matching phases from a typical subject are depicted in figure 2.

Supplementary Figures

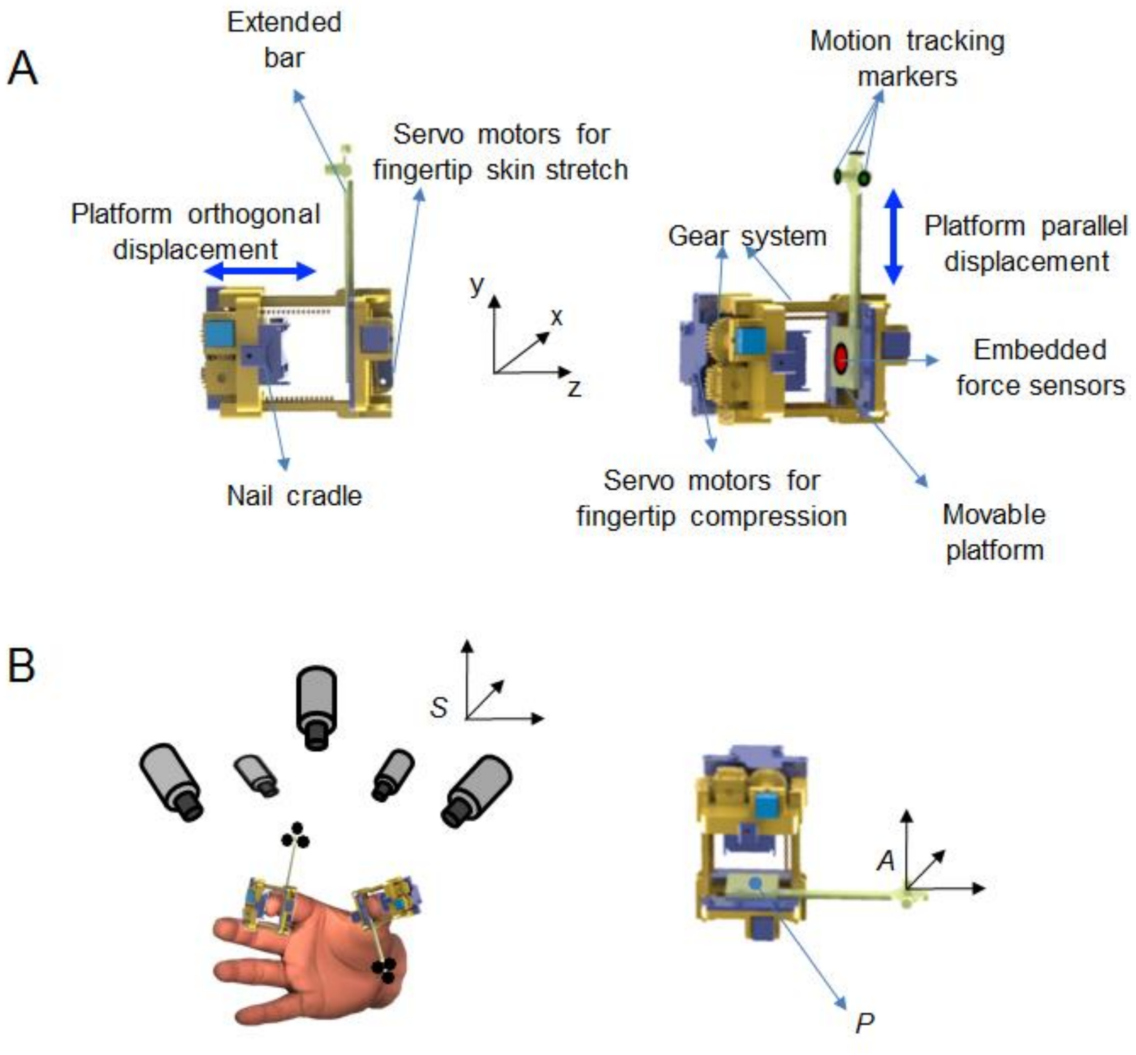

Figure S1. *Apparatus.* A) Wearable haptic devices used in Experiment 2. B) Integration of the wearable haptic device with the motion tracking system (see *center of pressure calculation* in Supplementary text).

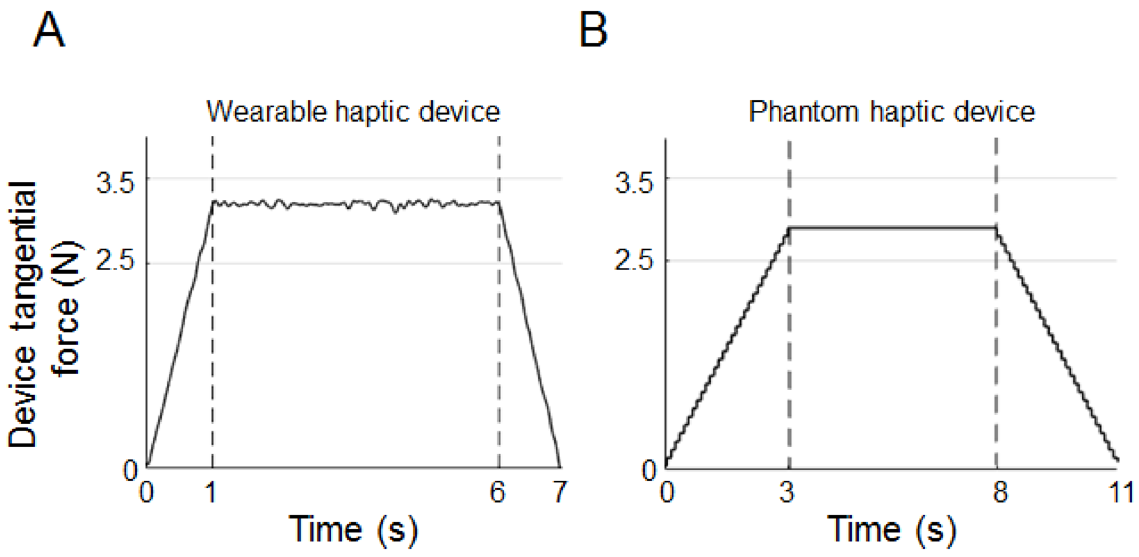

Figure S2. *Devices force profiles.* A) Tangential force profile produced by the haptic device used in Experiment 2. The force profile shown was obtained in separated tests aimed to assess the device reliability of producing consistent target force for 5 s. Device tangential force was recorded by an ATI nano 17 force sensor placed between the two platforms of the device. B) Tangential force profile produced by the Phantom device used in Experiment 3 (see *Methods* in Supplementary Material text).

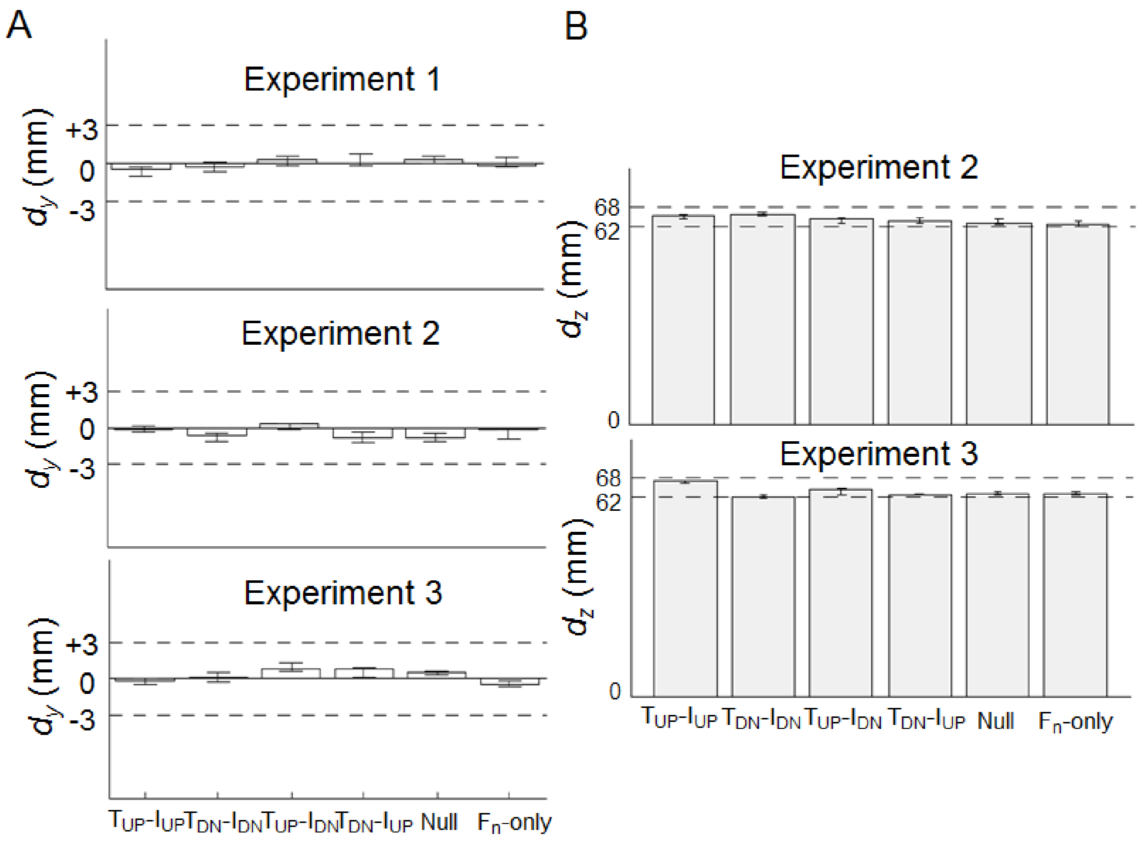

Figure S3. *Verification of compliance with experimental requirements* (see also *data analysis* in Supplementary Material text). A) Mean vertical digit distance (d_y) across all subjects measured during constant force production (Experiments 1 and 3) and finger pad stimulation (Experiment 2) throughout the "*Sense distance*" phase. B) Mean horizontal digit distance (d_z) from all subjects measured during the "*Sense distance*" phase during finger pad stimulation (Experiment 2) and digit force production (Experiment 3). In all plots error bars denote standard deviation**.**

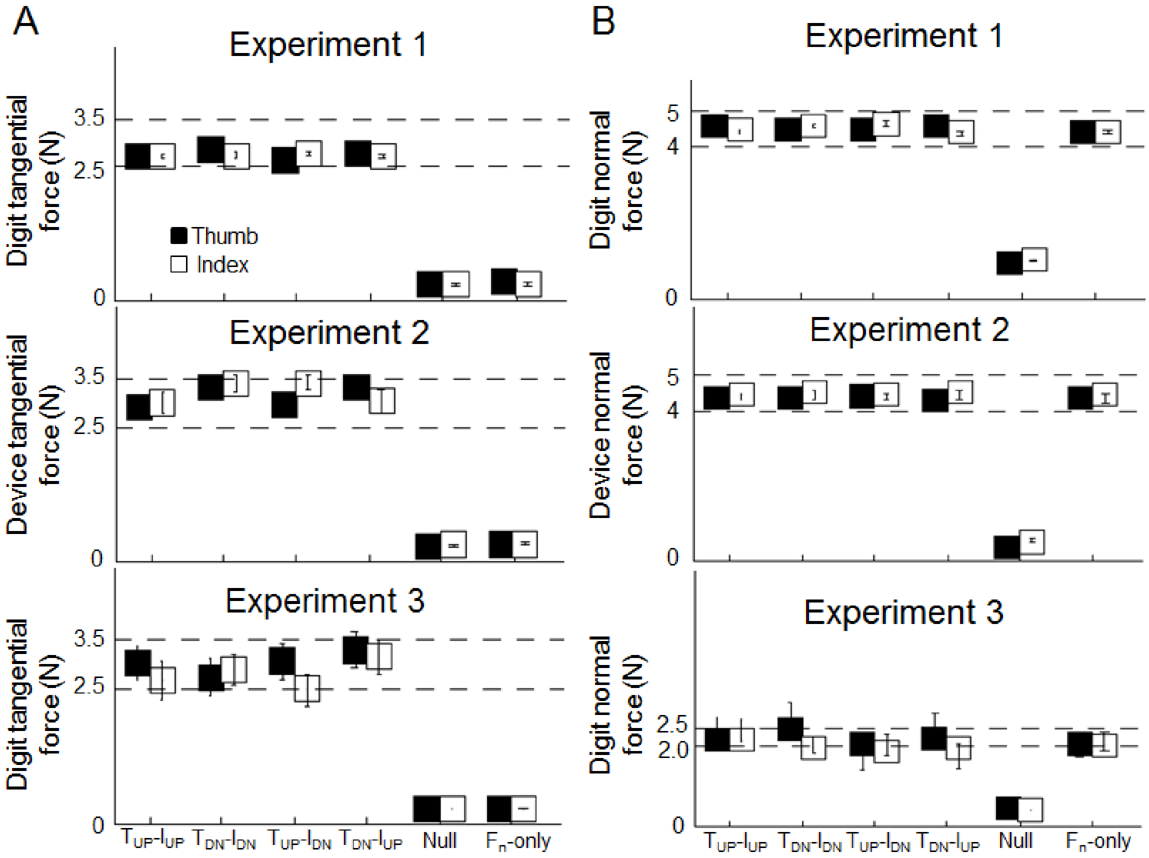

Figure S4. Digit forces in all experiments. Mean tangential (A) and normal (B) digit forces across all subjects exerted (Experiments 1 and 3) and stimulated by the wearable haptic device (Experiment 2) during the "*Sense distance*" phase (see *Data analysis* in Supplementary Material text for details on the calculations). Error bars denote standard deviation.

References

Shibata, D., Kappers A.M., Santello, M. 2014. Digit forces bias sensorimotor transformations underlying control of fingertip position. *Front Hum Neurosci* **8**, 564

Conti, F, Barbaglia, F., Morris, D., Sewell C. 2005. CHAI an open-source library for the rapid development of haptic scene. *IEEE World Haptics Conference*.
